# Modulation of tumor-associated macrophage activity with radiation therapy: a systematic review

**DOI:** 10.1007/s00066-023-02097-3

**Published:** 2023-06-22

**Authors:** Carlotta Becherini, Andrea Lancia, Beatrice Detti, Sara Lucidi, Daniele Scartoni, Gianluca Ingrosso, Maria Grazia Carnevale, Manuele Roghi, Niccolò Bertini, Carolina Orsatti, Monica Mangoni, Giulio Francolini, Simona Marani, Irene Giacomelli, Mauro Loi, Stefano Pergolizzi, Elisabetta Bonzano, Cynthia Aristei, Lorenzo Livi

**Affiliations:** 1 Radiation Oncology, Azienda Universitaria Ospedaliera Careggi, Università degli Studi di Firenze, Largo Brambila 1, 50134 Florence, Italy; 2 Radiation Oncology Unit, Fondazione IRCCS Policlinico San Matteo, Pavia, Italy; 3 Radiation Oncology, Santa Chiara Hospital, Trento, Italy; 4 Proton Treatment Center, Azienda Provinciale per i Servizi Sanitari, Trento, Italy; 5 Radiation Oncology Section, Perugia General Hospital, 06129 Perugia, Italy; 6 Department of Experimental and Clinical Biomedical Sciences Mario Serio, University of Florence, Florence, Italy; 7 Radiation Oncology Unit—Department of Biomedical, Dental Science and Morphological and Functional Images, University of Messina, Messina, Italy

**Keywords:** Immunomodulation, Radiotherapy, Macrophages, Radiobiology, Cancer

## Abstract

**Objective:**

Tumor-associated macrophages (TAMs) are the most represented cells of the immune system in the tumor microenvironment (TME). Besides its effects on cancer cells, radiation therapy (RT) can alter TME composition. With this systematic review, we provide a better understanding on how RT can regulate macrophage characterization, namely the M1 antitumor and the M2 protumor polarization, with the aim of describing new effective RT models and exploration of the possibility of integrating radiation with other available therapies.

**Methods:**

A systematic search in line with the Preferred Reporting Items for Systematic Reviews and Meta-Analyses (PRISMA) guidelines was carried out in PubMed, Google Scholar, and Scopus. Articles from January 2000 to April 2020 which focus on the role of M1 and M2 macrophages in the response to RT were identified.

**Results:**

Of the 304 selected articles, 29 qualitative summary papers were included in our analysis (16 focusing on administration of RT and concomitant systemic molecules, and 13 reporting on RT alone). Based on dose intensity, irradiation was classified into low (low-dose irradiation, LDI; corresponding to less than 1 Gy), moderate (moderate-dose irradiation, MDI; between 1 and 10 Gy), and high (high-dose irradiation, HDI; greater than 10 Gy). While HDI seems to be responsible for induced angiogenesis and accelerated tumor growth through early M2-polarized TAM infiltration, MDI stimulates phagocytosis and local LDI may represent a valid treatment option for possible combination with cancer immunotherapeutic agents.

**Conclusion:**

TAMs seem to have an ambivalent role on the efficacy of cancer treatment. Radiation therapy, which exerts its main antitumor activity via cell killing, can in turn interfere with TAM characterization through different modalities. The plasticity of TAMs makes them an attractive target for anticancer therapies and more research should be conducted to explore this potential therapeutic strategy.

## Background

Solid tumors are composed of both malignant cells and several nonmalignant hematopoietic and mesenchymal cells. Among the latter are tumor-associated macrophages (TAMs), which represent the most abundant subpopulation of tumor-infiltrating immune cells in the tumor microenvironment (TME) [1] and can interfere with tumor progression and neoangiogenesis. TAMs are extremely plastic immune cells, with two polarized states: classically activated M1 and alternatively activated M2 macrophages [2]. M1 macrophages play critical roles in innate host defense by producing reactive oxygen/nitrogen species (ROS/RNS) and proinflammatory cytokines such as interleukin (IL)-1β, IL‑6, and tumor necrosis factor α (TNF-α). In terms of their activity, they are generally considered as antitumor macrophages [3]. On the other hand, cytokines such as IL‑4, IL-10, and IL-13 can induce macrophage polarization to the M2 subtype, which is not only crucial for the onset of the classical Th2 immune response (i.e., humoral immunity, wound healing, tissue remodeling), but it is also key for the production of anti-inflammatory cytokines such as IL-10 and TGF‑β which foster tumor evolution. M2 macrophages are therefore considered to be protumor cells [4]. However, this “black and white” model has shown its limitations, mainly due to the existence of multiple intermediate states between M1 and M2; the polarization process is therefore dynamic, and macrophages often display characteristics of both profiles at the same time.

Besides its cytocidal effect on cancer cells, radiotherapy (RT) also plays a role in affecting the TME through multiple mechanisms, both direct and indirect, acting on different cell types. The interaction with tumor vascularization and immune cells remains crucial [5]. Endothelial damage, a central player in the induction of therapy-related inflammation, hampers CD8+ T cell infiltration into tumors and promotes the development of an immunosuppressive milieu affecting the efficacy of cancer therapies. As a consequence, suppressor cells such as M2 TAMs, myeloid-derived suppressor cells (MDSCs), and regulatory T cells (Tregs) gather together. Furthermore, hypoxic regions within the tumor are increased and hinder oxygen-dependent DNA damage, providing an even more reduced anticancer RT effect. We believe that a better understanding of the processes underpinning macrophage characterization under the influence of irradiation (IR) could be of help to establish new, effective RT schemas. Moreover, the association of RT with other available treatment options (i.e., immunotherapy) should be explored.

## Methods

### Search strategy

A systematic search in line with the Preferred Reporting Items for Systematic Reviews and Meta-Analyses (PRISMA [6]) guidelines was carried out in PubMed, Google Scholar, and Scopus. It was performed from January 2000 to April 2020 in order to identify articles focused on the role of M1 and M2 macrophages in response to radiation. Medical terms referring to radiotherapy were used in combination with M1 and M2 (note that we used the “AND” Boolean logic symbol to restrict the area of investigation as follows: “macrophages and radiotherapy,” “radiation oncology,” “TAMs,” “TAM,” and “MERT”).

### Screening process

**Fig. 1:**
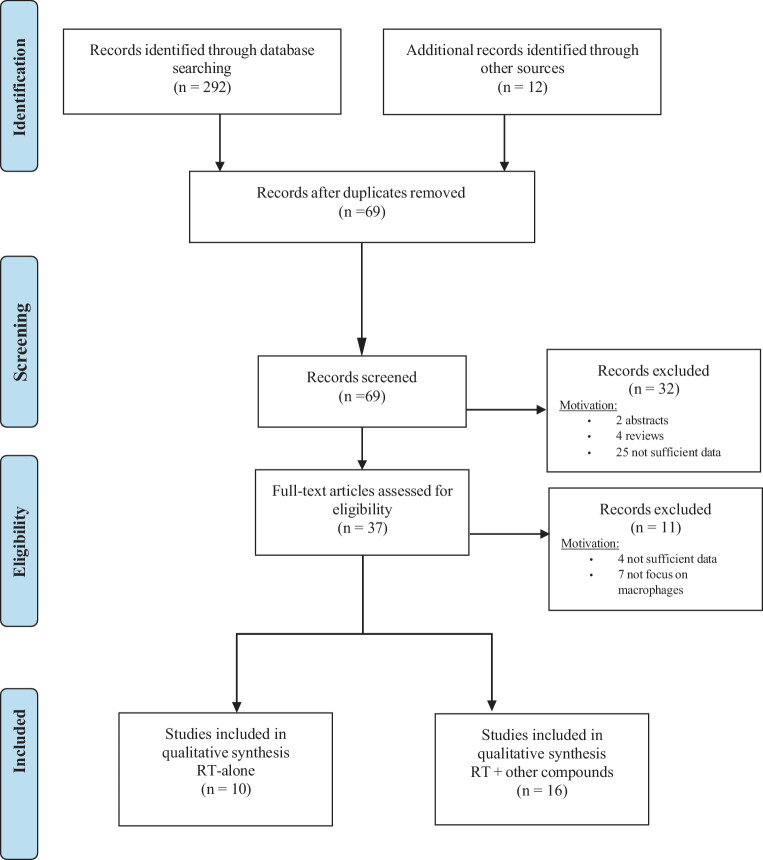
Flowchart of inclusion of studies in the systematic review. *RT* radiotherapy (From: [6]. For more information, visit www.prisma-statement.org)

All articles were screened by two independent reviewers (CB and MS). The reviewing selection process was based on title, abstract, and full text. Manuscripts exploring the role of and changes in M1 and M2 macrophages following irradiation were included in the systematic review. All study designs, with the exception of reviews, editorials, case studies, and conference abstracts/posters, were included. Languages other than English were excluded, as were studies that were not available in full-text version format. However, titles and abstracts selected by either one of the reviewers were included for additional screening. At each level of the screening process, when different opinions existed among the two reviewers as to whether to include a record or not, a mutual agreement was reached (see Fig. 1 for the flowchart). We extracted data from the included studies: for each paper, the principal author, publication year, number of patients, age, type of diagnostic imaging, treatment, and outcomes of interest were recorded. Data were summarized in evidence tables and described in the text.

## Results

Of the 304 articles initially screened and considered potentially relevant to the topic of this study, 26 were eventually included. The selected articles were categorized according to the description of M1/M2 changes when exposed to radiotherapy. The research flow and selection process are shown in Fig. 1. We separately analyzed the manuscripts containing only radiation therapy (10 articles) and those that explore the impact of radiotherapy and concomitant drugs on macrophage status (16 articles). For convenience, based on different IR doses analyzed, we referred to high-dose irradiation (HDI) as doses higher than 10 Gy, moderate-dose irradiation (MDI) as doses ranging from 1 to 10 Gy, and low-dose irradiation (LDI) as doses lower than 1 Gy.

### Role of TAMs in tumor initiation and progression

TAMs actively participate in tumor angiogenesis, matrix remodeling, invasion, immunosuppression, metastasis, and chemoresistance in various types of cancer. Several clinical studies have indicated that the presence of a tumor infiltrate characterized by high levels of TAMs represents a negative prognostic factor, as in the case of hepatocellular, ovarian, cervical, and breast cancer [7]. TAMs exhibit a wide spectrum of phenotypes, loosely categorized as the M1–M2 polarization spectrum, with M1 macrophages being generally proinflammatory and M2 macrophages presenting with anti-inflammatory and proangiogenic features. The activation of a particular macrophage profile seems to be dependent on the cytokine milieu, the production of specific growth factors, and the presence of hypoxia. While TAMs are very frequently differentiated into the M2 phenotype, the polarization process is, by definition, dynamic, and these cells very often display characteristics of both states at the same time.

### Effects of RT alone on macrophages status

Macrophages are one of the most radioresistant cell types [8]. This characteristic is attributed to the production of antioxidative molecules, such as manganese superoxide dismutase (MnSOD), which are responsible for cellular resistance against damaging effects produced by radiation-induced radicals such as reactive oxygen and nitrogen species (ROS and RNS, respectively). Tsai and colleagues [8] reported that after irradiation, Arg‑1, COX‑2, and inducible nitric oxide synthase (iNOS) are overexpressed in TAMs, which stimulates tumor growth. Of note, iNOS has a dual effect on tumor expansion, depending on its levels [9, 10]: the amount produced by M1 macrophages can kill cancer cells, while at lower concentrations, enough nitric oxide (NO) is produced to ensure a vasodilative effect and an increase in blood flow within the tumor, promoting its growth [11]. However, the iNOS pathway with the substrate l‑arginine—the one responsible for the cytotoxic effect—is blocked in M2 macrophages and replaced by the synthesis of ornithine and polyamines, which favor tumor cell proliferation. Several other cytokines secreted by TAMs, such as epidermal growth factor (EGF), TGF‑β, platelet-derived growth factor (PDGF), and basic fibroblast growth factor (bFGF), also have pro-proliferative actions. As a matter of fact, the presence of TAMs within the tumor leads to faster and increased growth of the neighboring tumor cells; in the post-irradiation setting, the production of tumor necrosis factor (TNF) by activated macrophages may favor the synthesis of protective proteins against subsequent killing by oxidative stress [12]. Another consequence of IR exposure is the massive recruitment of myeloid cells to the tumor site, which is thought to be a trigger for tumor regrowth after local irradiation. In this scenario, the inhibition of CSF‑1 receptor (CSF-1R) with PLX3397, a small molecule that blocks its tyrosine kinase activity, may enhance the cytotoxic effect of concomitant IR by preventing IR-recruited myeloid cells differentiating into protumor macrophages. In the in vivo study by Stafford et al. [13], combined IR and PLX3397 therapy was compared with IR alone in two different human GBM xenograft models. Median survival was significantly improved in mice receiving the combined approach.

The main studies exploring radiotherapy’s effects on macrophages are collected in Table 1.

**Table 1:** Studies exploring radiotherapy’s effects on macrophages

Authors (year)	Type	RT dose	Schedule	Tumor type	Cell type	Host species	Observation	RT effects
Park et al. (2019) [14]	Preclinical (in vitro/vivo)	14 Gy × 1 fr	RT → mice sacrificed at 2, 4, 8, 16, and 24 weeks after thoracic RT → BAL collected	None	Mouse lung MΦ MH‑S cell line and MLE12	Female C57BL/6N mice	RT promotes EMT in lung epithelial cells by TGF-β-producing M2 MΦ	↑ CCL2 production in BAL ↑ migration of MH‑S MΦ ↑↑ mRNA expression: Arg‑1 and CD206 ↑ IL-10 and TGF‑b
Choi et al. (2018) [15]	Preclinical (in vitro/vivo)	8 Gy × 1 fr; 20 Gy × 1 fr	–	Colon	CT26 cells	eC57BL/6 Tie2-Cre, Trp53flox/flox, Tgfbr2flox/flox, LSL-KrasG12D mice	EC-Trp53 deletion ↓ endothelial CXCR4 expression and M2 polarization of SDF-1+ TAMs after 20 Gy × 1 fr. The effetcs of RT-induced vascular damage on TAM polarization suggest that high-dose RT (~ 10 Gy) can evoke stronger tumor immune responses than low-dose RT	↑ CXCR4 in radioresistant tumor → ↑↑ SDF-1+ TAM recruitment and M2 polarization of TAMs ↑↑ population of iNOS+F4/80+ TAMs (M1-type) in WT tumors
Shen et al. (2018) [16]	Preclinical (in vitro/vivo)	5 Gy × 5 fr (consecutive days)	–	Lung	A549 (CLL-185™) and H157 (CRL-5802™); radioresistant subline cells: A549R26‑1 and H157R24‑1	NCI	IL-6-CCL2/CCL5 signaling triggers and ↑ MΦ migration to radioresistant cells, suggesting that the IL‑6 secreted by THP‑1 cells may be one of the major cytokines that trigger MEK/Erk activation in radioresistant lung cancer cells	↑ There were more cells expressing the F4/80 mouse MΦ marker in the tumors derived from A549R26‑1 cells compared to the tumors derived from A549P cells
Genard et al. (2018) [17]	Preclinical (in vitro)	0 to 10 Gy × 1 fr (proton)	Day 0: THP‑1 monocytes were differentiated into M2 MΦ → day 4: IR. To obtain M1 or M0 MΦ, cells were seeded 3 or 2 days, respectively, before IR	None	Human monocytic cell line (THP-1–ATCC TIB-202)	None	Early radioresistance (16 h post-RT) at 10 Gy ++ in M1 phenotype. IR of M0, M1, and M2 MΦ with 5–10 Gy promotes the reprogramming of M0 and M2 MΦ towards an M1 (M2 → intermediate phenotype [nuclear translocation of NFκB p65] → M1)	M1 resistance → ↑ γH2AX and 53BP1 labeling RT 10 Gy M0: ↑↑ M1 markers (IL‑6 and IL-8); ↓ EGF mRNA expression (specific marker of M2 phenotype) RT of M2 and M1: ↑↑ in TNFα secretion
Kung et al. (2018) [18]	Preclinical (in vitro/vivo)	25 Gy × 1 fr	CD11b-positive TAMs were isolated from tumors at 1 weeks, 2 weeks, and 3 weeks post-RT	Prostate	TRAMP C‑1	C57BL/6J mice	The MΦ in tumors irradiated after 2 weeks aggregated in hypoxic tumor regions with high Arg‑I and COX‑2 expression levels, indicating that MΦ phenotypes were polarized towards the M2 type	Six miRNAs were highly expressed compared to the control (miR-207, miR-195, miR-214, miR-32, miR-669d, let-7d)
Wu et al. (2017) [19]	Preclinical (in vivo/vitro)	20 Gy × 1 fr	Inoculation → 2 weeks: RT 20 Gy → mice were killed when tumors in the control group exceeded 1000 mm^3^	Colorectal	THP1 cells and RAW264.7 cells; human colorectal HCT116 cells	NCI	In vitro treatment of MΦ with various doses of RT led to their activation toward a pro-inflammatory phenotype	RT: ↑ IRF5- and IRF5-dependent target genes (IL‑6, TNFα or IFN-γ) RT or IFN-γ: ↑ NOX2
Leblond et al. (2017) [20]	Preclinical (in vivo/vitro)	In vitro: 2 Gy or 8 Gy × 1 fr; in vivo: 4 Gy (every 2 days) × 3 fr	Implantation → day 14: euthanized (3 days after RT). Late post-RT animals → day 27: euthanized (16 days after RT)	GBM	GL261	GL261 GB-bearing mice	M2 MΦ remained radioresistant whatever oxygen concentration. M0 and M1 MΦ undergo radio-induced mitotic catastrophe (not apoptosis). M1 MΦ: most radiosensitive phenotype (react similarly in both normoxia and in hypoxia)	RT ↓ MΦ but favors an enrichment in M2 phenotype in GB
Teresa Pinto et al. (2016) [21]	Preclinical (in vitro)	2/6/10 Gy	Western blot analysis of caspase‑7 expression on non-irradiated (−) or irradiated (2, 6, and 10 Gy; +) MΦ (*n* = 3), upon 1, 6, and 24 h	Colorectal	Human monocyte-derived MΦ	None	The relation between RT and inflammatory response → depends on cell type analyzed, RT quality, (mainly) delivered dose. Low doses (max 12 Gy at ≤ 1.0 Gy/fr) → anti-inflammatory phenotype. Higher doses (1 fr ≥ 2 Gy, total doses ≥ 40 Gy) → pro-inflammatory effect	↑ proinflammatory MΦ markers (CD80, CD86, HLA-DR) ↓ anti-inflammatory markers (CD163, MRC1, VCAN, CCL2, IL‑6, IL-10) ↑ Bcl2 and Bcl-xL expression (anti-apoptotic proteins) ↑ RelB; ↑/= cRel RT ↑ NF-kB MΦ
Pinto et al. (2016)	Preclinical (in vivo/vitro)	2 Gy × 5 fr (10 Gy)	Day 0: hMDM plant → day 1–11: MΦ differentiation → day 11: cancer cell plant → day 14–18 RT (2 Gy × 5 fr) → 6 h after last fr: material collection	Colorectal	Human monocyte-derived MΦ (hMDM) with RKO or SW1463 cells	–	MΦ sensitize RKO to RT-induced apoptosis, while protecting SW1463 cells. Additionally, co-culture with MΦ increased mRNA expression of metabolism- and survival-related genes more in SW1463 than in RKO	Irradiated MΦ: ↑ pro-inflammatory TNF, IL6, CCL2, CCR7, and of anti-inflammatory CCL18
Prakash et al. (2016) [22]	Preclinical (in vivo/vitro)	2 Gy (gamma ray)	25-week-old RT5 mice were IR systemically twice with 2 Gy (week 25 + week 26) → week 29 analysis	Insulinoma	Raw 264.7; peritoneal MΦ	RT5 mice	RT supported the acquisition of M1 features such as iNOS expression in non-polarized MΦ, and it also caused a phenotypic switching in TAMs with downregulation of M2-associated proteins such as Ym‑1, Fizz‑1, or Arg‑1	RT: ↑ iNOS, NO, NFκBpp65, pSTAT3, and proinflammatory cytokines; ↓ p38MAPK
Klug et al. (2013) [23]	Preclinical (in vivo/vitro)	0.5/1/2/6 Gy × 1 fr	Day 0: 24-week-old RT5 mice were IR in pancreatic region → day 10: transferred 5 × 10^6^ activated tag-specific TCRCD8+ or TCRCD4+ effector T cells in mice → analysis	Insulinoma melanoma	TCRCD8+ or TCRCD4+ T cells; HLA-A2-positive human melanoma cell line MeWo	RT5 mice; NSG mice	LDI-induced vascular normalization, T cell recruitment, and tumor immune rejection in RT5 tumors require TAMs and are correlated with intraepithelial MΦ accumulation and reduced infiltration of Gr1+ myeloid cells	While transfer of IR-MΦ resulted in ↑ intratumoral accumulation of CD3+, CD8+, and CD4+ T cells, transfer of unirradiated MΦ had no effect on tumor T cell infiltration
Crittenden et al. (2012) [24]	Preclinical (in vivo/vitro)	20 Gy × 3 daily fr	Tumors were inoculated s.c. in C57BL/6 mice → 14–17 days → RT 20 Gy × 3 fr → 1 day or 7 days following the final dose of RT → flow cytometry	Pancreas	Raw264.7 monocyte/MΦ cell line; 4T1 mammary carcinoma cell line; Panc02 murine pancreatic adenocarcinoma cell line	C57BL/6 mice	Following RT in mice, there is an influx of tumor MΦ that ultimately polarize towards immune suppression. Using in vitro models, it was shown that this polarization is mediated by NFκB p50. Tumor MΦ polarization limits the efficacy of RT, and the expression of NFκB1 limits the efficacy of RT in vivo	RT: ↑ CD11b+ cells, F4/80+ MΦ; upregulation of ccl2 and ccl7; Raw264.7 MΦ ↓ their iNOS expression; tumor MΦ =/↑ a polarized M2 phenotype. ↓ B cell, T cell and endothelial markers. Raw264.7 MΦ ↑ Arg I
Tsai et al. (2007) [8]	Preclinical (in vivo/vitro)	25 Gy × 1 fr or 60 Gy × 15 fr/3 weeks or sham-irradiated when 4 mm in diameter	–	Prostate	TRAMP-C1	C57Bl/6J mice	Tumor-associated MΦ in the post-RT tumor microenvironment express higher levels of Arg‑1, COX‑2, and iNOS, and promote early tumor growth in vivo	↑ Arg‑1, COX‑2, and iNOS

*fr* fraction, *RT* radiotherapy, *BAL* bronchoalveolar lavage, *MΦ* macrophage, *EMT* epithelial-to-mesenchymal transition, *TGF‑β* transforming growth factor-beta, *CCL2* monocyte chemoattractant protein‑1 or MCP‑1, *mRNA* messenger ribonucleic acid, *Arg‑1* arginine‑1, *CD206* mannose receptor or MRC1, *IL-10* interleukin-10, *MRT* microbeam radiation therapy, *EC-Trp53* p53, also known as TP53 or tumor protein (EC:2.7.1.37), *CXCR4* C-X‑C chemokine receptor type 4 (also known as fusin or CD184, cluster of differentiation 184), *SDF‑1* stromal cell-derived factor 1 (also known as C‑X‑C motif chemokine 12, CXCL12), *TAMs* tumor-associated macrophages, *iNOS* inducible nitric oxide synthase, *WT* wildtype, *NCI* female nude mice, *THP‑1* human acute monocytic leukemia cell line, *MEK/Erk* mitogen-activated protein/extracellular signal-regulated kinase kinase, *NFκB p65* nuclear factor kappa-light-chain-enhancer of activated B cells p65 subunit, *γH2AX* phosphorylated H2AX, *EGF* epidermal growth factor, *COX‑2* cyclooxygenase‑2, *miRNAs* microRNA, *IRF5* interferon regulatory factor 5, *IFN‑γ* interferon gamma, *GBM* glioblastoma multiforme, *HLA-DR* human leukocyte antigen–DR isotype, *Bcl2* B-cell lymphoma 2, *Bcl-xL* B-cell lymphoma extra-large, *TCRCD8+-4+* activated tag-specific TCR transgenic CD8+ or CD4+

#### High-dose irradiation

Several authors have shown that HDI can expand the number of M2-like TAMs in the tumor milieu. In an in vitro experiment, 20 Gy irradiation of M1 Raw264.7 macrophages led to TAM repolarization toward a M2-like profile [24]. More specifically, IR can activate NFκB p50 and determine an increase in IL-10 levels and an inhibition of TNFα production. A subsequent in vivo experiment from the same group showed that HDI was able to recruit M2 macrophages to the tumor site [24]. Tsai et al. reported how levels of M2 TAMs can raise after delivering HDI (25 Gy single-fraction or hypofractionated RT up to a total dose of 60 Gy) to prostate cancer cells. The authors observed an upregulated mRNA expression of Arg1 and Cox‑2 in TAMs together with a low iNOS level, favoring both angiogenesis and tumor growth in a murine model [8]. Similar results in terms of M2 macrophage proliferation and subsequent release of proangiogenic molecules were obtained after irradiation of oral cancer cells with a single dose of 12 Gy [25].

HDI was also found to be responsible for an improvement in suppressor T cell activity in pancreatic cancer cells [26]. A decrease in production of iNOS and an increase in levels of Arg1, PD-L1 (which stimulated the T response), and IL-10 (causing lymphocyte anergy) were observed.

Enhanced activity of M2 TAMs following IR was also documented in a lung cancer model [14]. Notably, the radiation-induced endothelial damage caused augmented production of CCL2 in the BAL fluid, ultimately leading to M2 macrophage colonization and hyperexpression of Arg 1 and CD206 in the weeks following irradiation.

M2 TAM polarization is therefore also favored by radiation-induced tumor vascularization via the endothelial-to-mesenchymal transition (EndMT), as shown by Choi et al. [15]. Of note, authors suggest that HDI may elicit a stronger immune response than LDI, due to the rate of indirect tumor cell death resulting from vascular damage.

Overall, these results well describe how HDI polarizes TAMs in an M2-like phenotype and promotes their recruitment to the tumor site, eventually leading to induced angiogenesis and accelerated tumor growth.

#### Moderate-dose irradiation

MDI (i.e., 2 Gy × 5) can enhance a proinflammatory state in M1 macrophages. Classic proinflammatory markers such as human leukocyte antigen cell surface receptor (HLA-DR) and CD86 are upregulated, while anti-inflammatory molecules (which characterize the M2 phenotype) are hindered, with a reduced mRNA expression of CD163, C‑type mannose receptor 1 (MRC1), and CD206, and decreased IL-10 secretion.

Phagocytotic activity, typically associated with the M1-like phenotype, is enhanced by MDI, which, on the contrary, has no influence on the ability of cocultured macrophages to promote cancer cell invasion and angiogenesis (typically related to the M2-like profile) [27]. Ex vivo γ‑MDI of CD11b+/Gr‑1 peritoneal macrophages demonstrated augmented levels of iNOS [22] and proinflammatory activity was documented in both murine and human models after 2–4 Gy of γ‑irradiation, with increased levels of TNFα, IFNγ, IL‑6, and IL-1β mRNA expression.

Different from previous experiences which documented the activation of NFκB p65 for TAM reprogramming, Wu et al., in their research, underlined the crucial role of the kinase ATM in promoting the M1-like phenotype through the regulation of IRF5 expression [19].

Of note, in vitro studies clarified that MDI is capable of inducing an M1 phenotype in nonpolarized macrophages and enhancing this profile in those which are already polarized; on the contrary, moderate irradiation cannot reprogram M2 TAMs.

In their study, Pinto et al. [28] set up cocultures of unpolarized macrophages with both radiosensitive (RKO cells) and radioresistant colon cancer cells (SW1463 cells). In the first scenario, MDI (which consisted of a total dose of 10 Gy in five daily fractions) led to reduced mRNA expression of some proinflammatory markers (such as CCR7 and IL1β), with no changes documented for anti-inflammatory markers, while cancer cell invasion and migration were promoted. Interestingly, when macrophages were cocultured with radioresistant cells, both proinflammatory (CCR7, CD80) and anti-inflammatory markers (IL-10 and CCL18) were overexpressed, with no changes in cancer cell migration and invasion. According to these findings, unpolarized macrophages develop a different phenotype based on the type of cancer cells they interact with [28].

#### Low-dose irradiation

Local radiotherapy influences the activity of tumor-specific T cells through several mechanisms: it determines a higher rate of antigen release from dying tumor cells; it stimulates antigen-presenting cell subsets; and, lastly, it enhances T cell migration. In their experiments with human melanoma xenografts and human pancreatic cancer specimens exposed to LDI, Klug et al. wanted to assess whether local single irradiation of 0.5–2 Gy can be used as an adjunct strategy to improve the efficacy of multiple immunotherapeutic approaches [23]. The authors found out that, due to iNOS activity, the classic Th2 response was completely (IL‑4 and IL-13) or markedly (IL‑5, IL‑6, IL‑9, and IL-10) inhibited after tumor irradiation. Indeed, iNOS inhibition has been shown to restore the Th2 response even in irradiated tumors. The iNOS-expressing TAMs were repolarized by irradiation towards an M1-like profile, being responsible for vascular normalization, T cell proliferation, and an antitumor response. Therefore, the adoptive transfer of radiation-induced iNOS-expressing macrophages may represent a promising strategy to explore to potentiate classical immunotherapeutic approaches.

Furthermore, in specific murine models, LDI led to activation of p38 MAPK in macrophages, with an associated transitory increase in TNF‑α production [29]. After 15 min from the delivery of 0.5 Gy gamma radiation, the upregulation of MKP‑1 was responsible for inactivation of p38 MAP‑K with suppressed production of proinflammatory TNF‑α.

### RT administered with concomitant agents: effects on macrophages status

Table 2 includes a list of studies, mostly preclinical, which explore the role of concomitant administration of RT and immunomodulating drugs in terms of their influence on macrophage status.

**Table 2:** Studies exploring the effects radiotherapy plus concomitant agents on macrophages

Authors (year)	Type	RT dose	Concomitant agent	Schedule	Tumor type	Cell type	Host species	Observation	RT effects
Kim et al. (2020) [30]	Preclinical (in vitro/vivo)	4 Gy × 1 fr	HS-1793 (resveratrol analogue)	HS-1793 injection → after 24 h RT → after 24 h Hs-1793 (twice a week)	Breast	FM3A murine breast cancer cell line	Female C3H/He mice	HS-1793 inhibits infiltration of CD206+ TAMs in tumor tissue of irradiated tumor-bearing mice via upregulation of IFN‑γ	↑ Lymphocyte proliferation with concanavalin A ↓ No. Tregs, IL-10, TGF‑b; inhibited CD206+ TAM infiltration in tumor tissue (vs. RT alone)
Wan et al. (2020) [31]	Preclinical (in vitro/vivo)	20 Gy × 1 fr	Irradiated tumor cell-released microparticles (RT-MPs)	–	Lung	Murine LLC (lung), human A549 (lung), and other cancer cells	Male C57BL/6 J wild-type (WT) mice → pleural inoculation of LLC-LUC cells → MPE model	RT-MP-treated BMDM-M2 cells showed increased phagocytosis of LLC cells compared to that of control BMDM-M2 cells. ↑ Jak-STAT and MAPK pathway (M1-related). RT-MP-treated MΦ showed much stronger ability to phagocytose tumor cells	Following phagocytosis of RT-MPs: MΦ showed M1 polarization ↑↑↑ CD86, MHCII, and ↓ CD206 Upregulation of M1-mRNAs; ↑ iNOS; ↑↑↑ PD-L1 expression on the MΦ surface Downregulation of M2-mRNAs; ↓ CD206 in MPE mice (< *p* = 0.001)
Stessin et al. (2020) [32]	Preclinical (in vitro/vivo)	10 Gy × 1 fr	Anti-PD‑1 blockade (aPD-1)	Implantation → Day 10: RT 10 Gy/1 fr → anti-mouse PD‑1 antibody immediately after RT, and two more doses on day 12 and 14 post-tumor implantation	Astroglioma	GL261-eGFP (murine astroglioma with eGFP construct)	Immunocompetent C57BL/6 mice	Both CD8+ T‑cells and MΦ are necessary for the full effect of combined therapy, with T lymphocytes appearing to play a role early on and MΦ mediating a later phase of the combined treatment effect. RT stimulated M1 but not M2, increasing the M1/M2 ratio	↑ CD8‑α, granzyme‑b, and perforin‑1; PD-L1 and CTLA‑4; IFN‑β ↑↑ serum IFN‑γ RT alone: FoxP3; PD-L1 and CTLA‑4; RT alone elicited maximal changes in the expression of molecules important for the activation of immune cells, such as IFN‑γ, MHC-II (H2-ab1), CD74, and the costimulatory molecules CD40, CD80, and CD86
Liu et al. (2020) [33]	Preclinical (in vitro/vivo)	2 Gy × 1 fr; 4 Gy × 1 fr	Monophosp horyl lipid A (MPLA)	Injected MPLA 12 h before RT through intragastric administration → RT	–	GC‑1 spg cells mice spermatogonia; RAW264.7	Male WT C57BL/6 mice	↑↑ TLR4 pathway in MΦ after MPLA stimulation	DNA-PKcs T2609 and p‑ATR showed higher activation level ↓ γH2AX, Bax, and cleaved-caspase3
Shi et al. (2019) [34]	Preclinical (in vitro/vivo)	4 Gy × 1 fr	PI3Kα-selective inhibitor CYH33	Cells were treated with CYH33 alone or concurrently with RT (4 Gy) for 72 h	Esophagus	Esophageal squamous cancer cells (ESCC)	Female nu/nu athymic BALB/cA mice	Inhibition of PI3Kα also potentiated the activity of RT to inhibit the clonogenesis of ESCC	RT ↑ infiltration MΦ (++ M2), while CYH33 abrogated this process ↓ DNA repair gene set after RT and further ↓ in combination with CYH33
Chen et al. (2019) [35]	Preclinical (in vitro)	6 Gy × 1 fr	IL‑6	Tissues were cultured and immediately received RT → cultured at 37 °C in humidified incubator (24 h) → tissues were rinsed with fresh PBS and frozen	HNSCC	HPV16+ HNSCC cell lines SCC47 and SCC104 and HPV-cell lines CAL33 and SAS	–	HPV stimulates IL‑6 secretion that promotes polarization of MΦ to an M1 subtype. M1 MΦ enhances radiation-induced DNA damage	M1 MΦ were significantly more prevalent in HPV+ HNSC. IL-6-induced M1 MΦ polarization. M2 MΦ were less abundant in HPV+ compared to HPV-HNSCC
Tabraue et al. (2019) [36]	Preclinical (in vitro/vivo)	Gamma irradiation (1 Gy/min) up to 10 Gy	LXR ligand GW3965 and synthetic LXR antagonist GSK144023 3A (denoted as GW233)	Cells were seeded + incubated overnight with growth medium → RT → cells were incubated at 37 C and 5% CO_2_	–	Primary and immortalized murine bone marrow derived MΦ (BMDM)	WT and LXRa/b-deficient (Nr1h3−/−, Nr1h2−/−) mice	Inhibition of LXR activity or LXR-deficient MΦ display ↑↑ in RT-induced proinflammatory markers and concomitant ↓ in some M2 markers	LXR-deficient MΦ exposed to RT: ↑ protein levels of γ‑H2AX and p53; ↑ cell membrane damage, and ↓ cell viability LXR deficiency: ↑ caspase‑1 activation + LDH release in BMDM; ↑↑ expression of proinflammatory markers (IL-1β, IL-6) + iNOS in irradiated MΦ
Rafat et al. (2018) [37]	Preclinical (in vitro/vivo)	20 Gy × 1 fr	In T cell depletion: anti-CD4 and/or anti-CD8a Local CCL4-blocking: aCCL4 or isotype control	–	Triple-negative breast cancer	Luciferase-labeled 4T1 mouse mammary carcinoma; MDA-MB-231 human breast cancer parental cells; MEFs	Female BALB/c (4T1 only) or Nu/Nu (4T1, MDA-MB-231) mice	RT-induced increase in MΦ infiltration in the absence of CD8þ T. These results suggest that normal tissue RT response may facilitate tumor cell invasion and recurrence in higher-risk patients with low lymphocyte counts following RT	MΦ CCL4 secreted in the MFP attract circulating tumor cells In vivo, CCL4 blocking antibody in MFP ↓↓ tumor cell recruitment to irradiated MFPs
Rahal et al. (2018) [38]	Preclinical (in vitro)	0, 2, 4, or 6 Gy	IL4/IL13; PM37 (phosho-STAT6 inhibitor)	THP-1 + primary human monocytes → differentiated into MΦ and polarized (M1/M2) → cocultured with IBC cells (24 h) → RT	IBC	IBC cells: SUM149, KPL4, MDA-IBC3, or SUM190	–	Inhibition of M2 polarization by PM37 can prevent radioresistance of IBC by downregulating PRKCZ	Expression of M2 polarization markers ↑ in M2-polarized MΦ after treatment with IL4/IL13 compared to M0 or M1-polarized MΦ. Pretreating M2-THP1 MΦ with PM37 ↓ the radioresistance induced in IBC cells after coculture
Yu et al. (2017) [39]	Preclinical	4 Gy × 1 fr (in vitro)	β‑elemene	–	Lung	Mouse RAW264.7 MΦ; mouse Lewis lung carcinoma cells	–	β‑elemene regulated the polarization of MΦ from M2 to M1. β‑elemene also inhibited the proliferation, migration, and invasion of lung cancer cells and ↑ radiosensitivity	After treating with β‑elemene: ↑ iNOS (M1 marker) and ↓ Arg‑1 (M2 marker)
Seifert et al. (2016) [40]	Preclinical (in vitro/vivo)	*Pancreas* → 2–12 Gy *Mice* → 6 Gy × 3 fr (48 h intervals). *Chemo‑RT experiments* → hypofractionated RT (12 Gy)	CSF1 (or MCSF) or F4/80	–	PDA	FC1242 cells derived from pancreas of KPC mice	p48Cre; LSL-KrasG12D (KC) and p48Cre; LSLKrasG12D; LSL-Trp53R172H (KPC) mice C57BL/6 mice	RT exposure causes MΦ in PDA to acquire an immune-suppressive phenotype and ↓ T cell antitumor responses. RT-induced M‑CSF expression in tumor cells drives TAM infiltration and M2-polarization	Pancreas from mice exposed to RT: ↑ numbers of CD4+ T cells, T‑helper 2, T‑regulatory cell phenotypes and ↓ CD8+ T cells
Stafford et al. (2016) [13]	Preclinical (in vitro/vivo)	12 Gy WBI	PLX3397 (inhibitor of CSF-1R’s tyrosine kinase activity)	U251-luc tumors + GBM12 tumors implanted in mice → mice were treated: PLX3397 alone (40 mg/kg/d), RT alone, or RT + PLX3397	GBM	U251-luc tumors and GBM12 tumors	Athymic nu/nu (nude) mice	CSF-1R inhibition blocks differentiation of protumorigenic TAMs that contribute to GBM recurrence	RT: ↑ CSF‑1 and IL-34 in GBM12 tumors; ↓ IL6 PLX3397: ↑ IL6, NOS2, TNFα (expression of pro-inflammatory M1) RT + PLX3397:
Okubo et al. (2016) [25]	Preclinical (in vitro/vivo)	12 Gy × 1 fr	IFN-γ/LPS and IL-4/IL-13 stimulation	OSCC xenograft mouse model using OSC-19 cells → implanted tumors grew progressively → RT 12 Gy → followed by regrowth	OSCC	OSC-19 and HSC‑3 cell lines; WEHI274.1 cell line	Female 4–7-week-old BALB/c nude mice	Local RT → vascular damage + hypoxia in the tumor and ↑ infiltration of CD11b myeloid cells with characteristics of M2Mφs; ↑ vascularization + tumor progression after RT	After RT, recurrence: ↑ IL-13Rα2+ cells in CD11b+ cells in tumors grown ↑ CD206+ M2Mφs
Ashcraft et al. (2015) [41]	Preclinical (in vivo)	5, 6, 7.5, 9 or 10 Gy × 5 fr	MnBuOE	FaDu cells were injected → 1 week: MnBuOE started, continued for 40 days → when tumor volumes reached 200–300 mm^3^: mice were randomized into RT dose groups	Hypopharyngeal carcinoma	1 × 10^6 FaDu cells → xenograft	C57Bl/6 mice	MnBuOE has radioprotective and radiosensitizing properties in normal tissue vs. tumor, respectively	MnBuOE: ↑↑ CD68+ MΦ. In particular, there was a 35% ↑ in the number of CD80+ M1 MΦ in the 5 × 5 Gy group compared with saline + RT
Ridnour et al. (2015) [42]	Preclinical (in vitro/vivo)	10 Gy × 1 fr	L‑NAME; DETA/NO; guanylyl cyclase inhibitor ODQ ODQ	Tumor cells were injected → grown for 1 week → palpable tumors (∼ 200 mm^3^) → day 7: RT 10 Gy ± post-RT L‑NAME (or aminoguanidine)	Squamous cell carcinoma	Jurkat clone E6‑1; ANA‑1 MΦ cell line; SCC or HT29 tumor cells	Female C3H/hen or athymic nude mice	Post-RT NOS inhibition improves radiation tumor response via Th1 immune polarization within the tumor microenvironment	Post-RT L‑NAME extended the RT-induced tumor growth delay only in syngeneic but not nude mice Cytotoxic Th1 cytokines (IL2, IL12p40, IFNγ) as well as activated CD8+ T cells ↑ in tumors receiving post-RT L‑NAME
Ager et al. (2015) [43]	Preclinical (in vitro/vivo)	6 Gy × 3 fr (consecutive days)	Anti-MMP14 inhibitory antibody (DX-2400) iNOS inhibitor	A single 4T1 primary tumor per mouse was established → local RT began 4 days after tumors reached approximately 40 mm^3^	Breast Cancer	4T1 cells; E0771 cells	NCr-*nu/nu* (nude) mice; BALB/c, C57BL/6 and* NOS2−/− *mice	X‑2400 inhibited tumor growth compared with IgG control treatment, ↑ MΦ numbers, and shifted the MΦ phenotype towards antitumor M1-like	The shift of MΦ phenotype towards antitumor M1-like is associated with a reduction in active TGFβ and SMAD2/3 signaling

*fr* fraction, *RT* radiotherapy, *BAL* bronchoalveolar lavage, *MΦ* macrophage, *EMT* epithelial-to-mesenchymal transition, *TGF‑β* transforming growth factor-beta, *CCL2* monocyte chemoattractant protein‑1 or MCP‑1, *mRNA* messenger ribonucleic acid, *Arg‑1* arginine‑1, *CD206* mannose receptor or MRC1, *IL-10* interleukin-10, *MRT* microbeam radiation therapy, *EC-Trp53* p53, also known as TP53 or tumor protein (EC:2.7.1.37), *CXCR4* C-X‑C chemokine receptor type 4 (also known as fusin or CD184, cluster of differentiation 184), *SDF‑1* stromal cell-derived factor 1 (also known as C‑X‑C motif chemokine 12, CXCL12), *TAMs* tumor-associated macrophages, *iNOS* inducible nitric oxide synthase, *WT* wildtype, *NCI* female nude mice, *THP‑1* human acute monocytic leukemia cell line, *MEK/Erk* mitogen-activated protein/extracellular signal-regulated kinase kinase, *NFκB p65* nuclear factor kappa-light-chain-enhancer of activated B cells p65 subunit, *γH2AX* phosphorylated H2AX, *EGF* epidermal growth factor, *COX‑2* cyclooxygenase‑2, *miRNAs* microRNA, *IRF5* interferon regulatory factor 5, *IFN‑γ* interferon gamma, *GBM* glioblastoma multiforme, *HLA-DR* human leukocyte antigen–DR isotype, *Bcl2* B-cell lymphoma 2, *Bcl-xL* B-cell lymphoma-extra-large, *JAK-STAT* Janus kinase/signal transducers and activators of transcription (Jak-STAT), *MAPK* mitogen-activated protein kinase, *MHC* major histocompatibility complex, *HNSCC* head and neck squamous cell carcinoma, *HPV+* human papillomavirus-positive, *MEFs* primary mouse embryonic fibroblasts, *MFP* mammary fat pad, *IBC* inflammatory breast cancer, *PRKCZ* protein kinase C zeta, *PM37* phosphopeptide mimetic, *CSF1 or MCSF* neutralizing antibodies against macrophage colony stimulating factor 1, *PDA* pancreatic ductal adenocarcinoma, *CSF-1R* colony stimulating factor 1 receptor, *OSCC* oral squamous cell carcinoma, *MnBuOE* Mn(III) meso-tetrakis(N-n-butoxyethylpyridinium-2-yl)porphyrin, *L‑NAME* NOS inhibitor NG-nitro-l-arginine methyl ester

As reported by Zeng and colleagues [44], T lymphocytes have a key role in mediating the effects of stereotactic radiotherapy (SRT) and immunotherapy, while both macrophages and microglia are involved in a later phase. However, it has been hypothesized that the additional benefit of the combined approach relates to M1 macrophage-mediated effects [32]. In fact, the coadministration of radiotherapy and PD‑1 checkpoint blockade can boost the immune response by increasing the number of CD8+ lymphocytes and macrophages (namely the M1/M2 ratio).

The great majority of immunomodulating molecules that have been analyzed and reported in this review act by inhibiting the signaling pathways involved in M2 polarization and hinder M2 macrophage-mediated radioresistance. For example, PM37, a phosphopeptide mimetic targeting the SH2 domain of STAT6, was shown to decrease the expression of M2 polarization markers [38]. Pretreating macrophages with PM37 reduced the radioresistance they induced in inflammatory breast cancer (IBC) cells after coculture. In another paper by Shi et al. [34], the authors noted that combining radiation with PI3Kα inhibitors resulted in a synergistic activity against esophageal squamous cancer cells and patient-derived xenografts (PDX); more specifically, this association abrogated radiation-induced survival signals in both tumor cells and the tumor microenvironment, hindering M2-like macrophage infiltration.

## Discussion

The role of macrophages within the tumor milieu has gained a lot of interest in the recent literature; these immune cells show different phenotypic profiles according to the differently induced microenvironmental signals and cytokines [38]. This review captured the current knowledge about interactions between TAMs and radiation therapy.

Classically activated macrophages (M1) are mainly induced by Toll-like receptor ligands and Th1 cytokines, such as interferon IFN‑γ, while Th2 cytokines like IL‑4 and IL-13 can stimulate the adoption of an M2 profile (alternatively activated macrophages).

The acronym TAM usually refers to the M2-like phenotype, characterized by anti-inflammatory and protumoral activity. On the contrary, M1-like macrophages exhibit proinflammatory, phagocytic, and antitumor functions. M2 TAMs are responsible for augmented genetic instability, upregulated angiogenesis, and increased immunosuppressive signals, which favor metastatic spread. This cell profile is also associated with tissue remodeling and conditions characterized by augmented fibrosis, such as pulmonary fibrosis [4], due to the stimulated production of profibrotic molecules including TGF‑β, IGF‑1, and galectin‑3.

Multiple activated transcription factors and miRNAs regulate macrophages’ expression of a specific M1 or M2 phenotype. In particular, NFκB plays a key role: its active heterodimer NFκB (p50–p65) favors proinflammatory gene expression, such as TNFα, IL‑6, and IL1β, while the inactive homodimer NFκB (p50–p50) hinders the transcription process of such genes, ultimately leading to the anti-inflammatory profile which characterizes M2 macrophages [34].

In the setting of the tumor microenvironment (TME), where TAMs favor the epithelial-mesenchymal transition, these immune cells are indeed related to tumoral progression [1, 45]—both locally with enhanced tumor growth due to L‑arginine depletion [46–48] and systemically by increasing its metastatic potential. For all these reasons, TAM accumulation correlates with an unfavorable prognosis in many cancer types.

TAMs exert their immunosuppressive action by inhibiting T cell proliferation, thanks to the expression of specific molecules such as PD-L1 and PD-L2, which are inhibitory checkpoint regulators interacting with corresponding ligands on T cell membranes, ultimately leading to their inactivation [49, 50].

Furthermore, TAMs indirectly contribute to immunosuppression by producing chemoattractant molecules to recruit cells which further hamper the immune response, such as myeloid-derived suppressor cells (MDSCs), immature dendritic cells (DCs) and Tregs [51]. As M2 macrophages are activated by IL‑4 produced by CD4+ T cells, PMA/IL-4-treated THP‑1 cells are often used to generate TAMs [52, 53].

In this scenario, characterized by an intricate interaction between the TME immune environment and cancer cells, there is growing interest in the role of radiation.

Radiotherapy (RT) currently represents an essential component of the management of cancer patients, either alone or in combination with surgery or systemic therapies. The main goal of RT is to deliver a curative dose to the tumor while sparing the surrounding healthy tissues and organs.

**Fig. 2:**
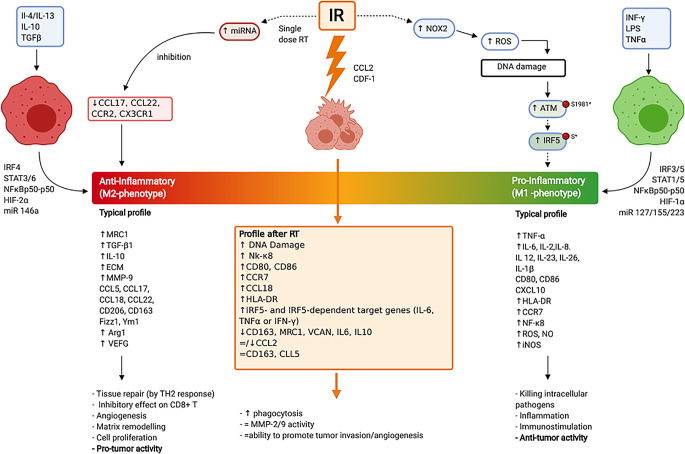
Schematic representation of radiation-induced effects on macrophages. TAMs within the tumor are either present as tissue-resident macrophages or are formed after circulating monocytes are recruited and subsequently differentiated. Soluble factors such as the chemokine ligand 2 (*CCL2*, also known as monocyte chemoattractant protein 1, MCP1), complement anaphylatoxins (C3a and C5a), and colony-stimulating factor 1 (CSF 1) are well-documented signaling molecules involved in the recruitment process. Furthermore, physical changes such as upregulation of HIF‑α subunits and damaging of the extracellular matrix leads to TAM infiltration and tumor cell proliferation. Polarization of monocytes into mature macrophages phenotypically falls into a wide spectrum of either inflammatory or immunosuppressive behaviors, depending on the expression of interleukins and lipopolysaccharides. *IR* irradiation, *miRNA* micro-ribonucleic acid, *CCL* CC chemokine ligand, *CC* CC chemokine receptor, *CX3CR1* C-X3‑C motif chemokine receptor 1, *IL* interleukin, *TGF* transforming growth factor, *IRF4* interferon regulatory factor 4, *STAT* signal transducer and activator of transcription, *NFkB p50/p50* nuclear factor kappa B subunit 1, *miR* micro-ribonucleic acid, *MRC1* mannose receptor C-type 1, *ECM* extracellular matrix, *MMP* matrix metallopeptidase, *CD206* mannose receptor, *CD163* cell-surface glycoprotein receptor member of the scavenger receptor cysteine-rich family class B, *Fizz1* resistin-like molecule alpha‑1, *Ym1* rodent-specific chitinase-like protein 3, *Arg1* arginase 1, *VEGF* vascular endothelial growth factor, *TH2* type 2 helper T, *DNA* deoxyribonucleic acid, *NF-kB* nuclear factor kappa light chain enhancer of activated B cells, *CD80* ligand for the proteins CD28, *CD86* cluster of differentiation 86, *HLA-DR* human leukocyte antigen–DR isotype, *IRF5* interferon regulatory factor 5, *IFN‑γ* interferon-gamma, *VCAN* versican, *NOX2* nicotinamide adenine dinucleotide phosphate (NADPH) oxidases 2, *ROS* oxygen-containing reactive species, *ATM* ataxia telangiectasia mutated, *CXCL10* C-X‑C motif chemokine ligand 10, *NO* nitric oxide, *iNOS* inducible nitric oxide synthase (Created with BioRender.com)

Alongside the killing effect on tumor cells, different RT doses may induce modifications of the local microenvironment that can affect tumor development. IR may enhance macrophage infiltration to tumor sites, accelerating tumor progression in several ways (summarized in Fig. 2,).

Wu et al. [54] demonstrated that different doses of IR can polarize macrophages to show a proinflammatory M1-like profile in xenograft tumor models and human rectal cancer specimens obtained from patients treated with neoadjuvant chemoradiation. This effect is attributed to the IR-induced activation of interferon regulatory factor 5 (IRF5): its mRNA levels and posttranslational modifications are regulated by ATM kinase, whose activation is not only decisive in radiation-elicited macrophage polarization, but which is also key for macrophage reprogramming after treatments with agents like cisplatin, γ‑interferon, and lipopolysaccharide. Furthermore, the authors demonstrated that upstream activation of NADPH oxidase 2 (NOX2)-dependent ROS, which is increased after IR exposure or IFN‑γ treatment, is also crucial for macrophages’ acquisition of a proinflammatory profile. The downregulation of this intricate pathway, at any level, can hinder macrophage activation towards a M1 phenotype, ultimately leading to poor tumor response after radiotherapy.

As demonstrated by Wang et al. [55], irradiation positively regulates IL‑6 levels; however, depletion of this cytokine was found to be associated with reduced macrophage infiltration after radiation exposure, indicating the crucial role of IL‑6 in this process.

Immunostimulating effects of IR in the tumor milieu include enhanced natural killer (NK) cell cytotoxicity and CD8+ infiltration, enhanced macrophage polarization towards an M1-like profile, reduced levels of Treg [56], and inhibition of the PD-1/PDL‑1 pathway [57].

Radioresistant tumors are characterized by a high level of macrophage infiltration, which contributes to the development of additional resistance to the cytotoxic activity of NK cells [58] through modification of tumor cell–NK cell interactions at specific ligand levels, namely PD-L1 and NKG2D [59]. It has been shown that NKG2D ligand expression on macrophages is upregulated upon coculture with NK cells [16].

Further studies will be crucial for revealing the role of THP‑1 CM in the alteration of NKG2D ligand levels (on tumor cells) and NKG2D receptor levels (on NK cells) in specific coculture systems including tumor cells, THP‑1, and NK cells. IL‑6, which is produced by THP‑1 cells, may be key in inducing tMEK/Erk activation in radioresistant cancer cells [60, 61]. Nevertheless, the role of other cytokines should also be taken into consideration for inducing tumor cells’ resistance to NK cell cytotoxicity (i.e., IL-10) [62].

In a murine model of breast cancer cells, Shiao and colleagues reported on how polarized Th2 macrophages and CD4+ T cells mediate tumor growth after radiation therapy, in part via suppression of CD8+ T cells [63]. More recently, Allen et al. [64] showed that macrophages can regulate the sensitivity of inflammatory breast cancer cells (IBCs) to radiation via increased production of IL‑6, IL‑8, IL-10, and protein kinase C zeta (PRKCZ), a previously reported modulator of radiosensitivity [65]. Irradiation promoted CT26 and 4T1 cells to secrete CCL2, which has a crucial role in recruiting TAM to the TME in a dose-dependent manner. Cheng et al. [66] observed that combining rosiglitazone treatment with irradiation significantly reduces the CCL2 level and its chemotactic effect responsible for TAM infiltration in irradiated tumors. Furthermore, the authors have highlighted that macrophage PPARc is a crucial mediator of the antitumor effect of rosiglitazone in vivo. Deletion of macrophage PPARc in mice not only facilitates tumor progression but also weakens the antitumor effects of PPARc agonists, with a concomitantly increased infiltration of CD11b+ myeloid cells and TAMs with proinflammatory and proangiogenic phenotypes [66].

TAMs, like other cells of the monocyte-derived DC system, have demonstrated phagocytic activity. A review was published recently focusing on TAMs’ phagocytic activity to improve innate anticancer immunity and promote T cell-mediated adaptive immune responses [67]. Interactions between tumor cells and TAMs that regulate phagocytosis are the result of “eat me” and “don’t eat me” signals [68]. Moreover, during radiochemotherapy treatment, there is an increased release of apoptotic tumor cells which favors activation of the efferocytosis pathway, which promotes anti-inflammatory function [69]. This process leads to rapid antigen degradation and therefore limits the cross-presentation capacity, ultimately promoting an immunosuppressive tumor microenvironment [70]. Comprehensive knowledge of these pathways will allow us to better identify targets, such as anti-CD47 and efferocytosis inhibitors, to modulate TAM phagocytic activity [67].

Macrophages can be considered rather radioresistant, as even high single doses have no significant impact on their viability, even though some hints toward increased DNA damage after exposure to ionizing radiation are found. In general, LDI seems to have a rather anti-inflammatory effect, while HDI seems to have a rather inflammatory impact on macrophage functionality. Cytokine secretion on the other hand is strongly dependent on various additional factors such as inflammatory background and radiosensitivity of the model, as well as on the applied dose.

## Conclusion

TAMs contribute to tumor progression in several ways, from enhanced genetic instability to induced metastasis formation and impaired protective adaptive immunity. The plasticity of these cells makes them an attractive target for anticancer therapies, which should have the goal of polarizing TAMs to the proinflammatory and tumoricidal side of the spectrum. Radiation therapy, which exerts its main antitumor activity via cell killing, not only enhances TAM recruitment to the tumor site, but can also interfere with their characterization in multiple modalities according to different doses and schedules of administration. Assessment of the microenvironment should be included in studies combining RT with systemic therapies, as an unexpected polarization could be detrimental to their synergy. More research should be conducted in the near future to explore this potential therapeutic strategy.
